# Barriers and Enablers for the Use of Digital Interactive Television in Nursing Home Settings: An Interview Case Study with Older Adults and Professionals

**DOI:** 10.3390/ijerph20031813

**Published:** 2023-01-18

**Authors:** Bérangère Naudé, Anne-Sophie Rigaud, Laila Kamali, Maribel Pino

**Affiliations:** 1 Faculté de Médecine, Université Paris Cité, Maladie d’Alzheimer, F-75013 Paris, France; 2 Broca Living Lab, CEN STIMCO, F-75013 Paris, France; 3 Service Gériatrie 1&2, Centre Mémoire de Ressources et Recherches Ile de France-Broca, AP-HP, Hôpital Broca, F-75013 Paris, France; 4 School of Medicine, Royal College of Surgeons, D02 YN77 Dublin, Ireland

**Keywords:** older adults, geriatric institutions, usability, digital interactive television, health technology assessment

## Abstract

Increasingly, public health programs are recommending the use of information and communication technologies to address the psychosocial needs of Older Adults (OAs). Recently, several applications that allow access to communication and stimulation functionalities using digital interactive television (DiTV) have been developed. The use of the television interface to access digital services seems to help meet several accessibility and usability needs of OAs. However, its use entails other challenges related to different dimensions (organizational, technological, ethical, etc.). This study aims to identify the factors that enable or hinder the use of DiTV by OAs living in geriatric institutions. A qualitative interview case study was conducted in three French geriatric facilities. A total of 25 semi-structured interviews were carried out with residents and care professionals, between February and April 2022, to identify enablers and barriers to DiTV use. Data were processed using a thematic deductive analysis inspired by a multidimensional Health Technology Assessment model. The analysis showed that DiTV use may be limited by organizational (e.g., workload), technological (e.g., ergonomic issues), human (e.g., health issues), ethical (e.g., privacy), and safety factors (e.g., frustration due to technical problems). A summary of these factors and five recommendations for DiTV implementation in geriatric settings are presented in this paper.

## 1. Introduction

Aging is accompanied by perceptual, physiological, and cognitive changes [1,2] that ultimately lead to a loss of functional independence in some Older Adults (OAs), and thus to their institutionalization. Social isolation is a central issue in Nursing Homes (NHs). Some authors [3,4,5] have estimated that 50–55% of elderly residents suffer from social isolation. With the COVID-19 pandemic and the successive lockdowns, OAs have seen the number of social activities drastically diminish (e.g., interruption of activities, decrease in visits from relatives) [6,7], increasing the risk of mental and physical health problems, such as depression, cardiovascular problems, etc. [8]. In recent years, several systematic literature reviews have shown the positive effect of various Information and Communication Technologies (ICTs) on quality of life, loneliness, and social isolation of OAs [9,10,11]. The term ICT refers to a wide range of tools such as computers, digital applications (e.g., medication management applications, email, messaging, and internet), social robots, telemedicine, video games, virtual or augmented reality applications, videoconference systems, or smartphones, among others. These reviews covered studies between 1970 and 2020 and highlighted the value of using technological innovations to support OAs living at home or in geriatric facilities. Multiple uses of these digital tools have been made, also proving their interest in the areas of rehabilitation and therapeutic follow-up of patients with specific health needs [12,13,14]. In recent years, public health policies in the field of aging have increasingly recommended the use of this type of technology-based interventions to meet the needs of the aging population [15,16].

However, the efficacy of technology-based Public Health Interventions (PHIs) can be greatly influenced by accessibility issues [11]. OAs often lack digital literacy and may experience difficulties using ICTs, which may limit technology acceptance in this population [17,18]. Digital literacy refers to the ability to use technological and digital devices and applications [17], this implies having the knowledge and skills required to use them. Aging is indeed accompanied by perceptual, physiological, and cognitive changes (e.g., decreased visual acuity, hearing loss, motor disorders, increased fatigability, slow behavioral responses, and reduced ability to process information [1,2]), which can negatively impact the use of technology [19]. As a result, the elderly population is more prone to encounter usability difficulties when using technological innovations than younger adults [20].

Usability problems can be even more critical among OAs living in care institutions, since admission into institutional care in this population is often motivated by the presence of important cognitive and functional limitations [21], that limit as well their capacity to use ICTs.

Digital literacy also depends on the willingness of OAs to use technological innovations. However, OAs often lack experience with ICT, making them feel anxious when confronted with technology use, thus unwilling to try digital innovations [17]. The use and acceptance of technological innovations by OAs can also be hindered by low self-efficacy, described by Bandura as “one’s capabilities to organize and execute the courses of action required to produce given attainments” [22]. Other critical factors for the acceptance and adoption of technologies by OAs are perceived ease of use and perceived usefulness, as described by the Technology Assessment Model (TAM) [23]. Thus, OAs usually prefer and benefit from easy-to-understand interfaces with a simple design and services that truly respond to their needs and/or interests [20].

Television (TV) has a central role in the everyday life of OAs (e.g., serving as a link to the outside world, limiting the feeling of social exclusion) [24], being used as a primary source of information and entertainment [25]. Many authors agree that the TV support offers OAs a much more familiar interface to access new functionalities, thus less anxiogenic, than more recent hardware applications (e.g., smartphones, computers, and tablets) [24,26,27,28,29]. Using familiar devices could decrease technology anxiety and help OAs to enter the digital era [17]. Over the past few years, the market for Digital interactive Television (DiTV) has significantly grown with the development and integration of new services, such as Internet access, video calls, shopping, or games, into the TV system [30,31]. However, the addition of these interactive features has forced users to adopt a more active role during their interaction with DiTV systems [32], creating new accessibility and usability issues for some population groups. Several studies carried out with OAs have already highlighted ergonomic problems related to DiTV interfaces (e.g., bad usability of the search functionality and user confusion when faced with the many services available on the DiTV) [30,33] as well as to the remote control use (e.g., unclear buttons’ labels and icons, and small buttons) [26]. Furthermore, the environment or context could also influence the acceptance of technological innovations by OAs. For example, in the case of the use of video calling technology in a NHs, OAs’ relatives and staff members (e.g., animator, technical manager, care staff members) usually set up the video calling device and initiate the video calls [34,35]. Thus, they play a key role in the use, and the related adoption, of technological innovations by OAs.

DiTV acceptance and use by OAs in NHs depend on a variety of factors (e.g., technological, ethical, organizational, social, etc.). Multidimensional approaches for Health Technology Assessment (HTA) could be useful to identify and analyze them. Such is the case of the European Health Technology Assessment model (EUnetHTA Core Model^®^, version 3.0) created by the international expert group of the European Network of Health Technology Assessment [36]. This multidimensional model could be used to evaluate and understand barriers that OAs may encounter when using DiTV systems.

Since telecommunication functionalities (e.g., video calls) seem to benefit OAs by increasing social activities and reducing loneliness [37,38,39], and DiTV systems appear to be more familiar to OAs than smartphones or tablets [24,26,27,28,29], further research about factors of use and acceptance of DiTV should be conducted. Baunstrup and Larsen [40] have carried out a series of interviews and a survey with OAs living alone in their own homes to identify barriers and reasons for the use of DiTV. However, the factors influencing the use of a DiTV system in NHs populations have not yet been investigated. This question seems interesting to explore, considering that DiTV is an easily generalizable technological tool that can bring benefits in terms of socialization and well-being in OAs, which could be further promoted by public health programs.

This paper presents a case study that aims to identify the barriers and enablers (human, technological, organizational, etc.) for the use of DiTV, reported by elderly NHs residents and care professionals of these facilities, using a multidimensional HTA model.

Results from this study would be useful to identify action levers to improve the implementation of the DiTV system and the telecommunication functionalities (i.e., messaging and video calling), by OAs in NHs.

## 2. Materials and Methods

### 2.1. Research Design

A qualitative case study using semi-structured face-to-face interviews was adopted for this study. For the purposes of this research, the case study approach was chosen taking into consideration that the DiTV system is a relative new service in French NHs and only a few institutions currently provide it. The participants were OAs and care professionals living or working, respectively, in NHs. Face-to-face interviews had the advantage of providing the interviewer, in addition to the person’s speech, with additional information, such as the intonation or non-verbal language that may be useful for the interpretation of the answers [41].

### 2.2. Participants

Three French NHs that have been equipped with a DiTV system were approached between February and April 2022. This study included OAs living in these facilities and care professionals who worked there.

Concerning the population of OAs, 18 residents were interviewed (11 women and 7 men) aged between 68 and 90 years (mean age 72.6 years). The inclusion criteria for OAs were to be living in a NH, to have a DiTV installed in their rooms, and to be able to participate in a 30-minute interview. Exclusion criteria included acute disease and severe cognitive, hearing, or vision impairment. For this purpose, the care staff, from participating facilities, established a list of potentially interested residents who met the inclusion criteria. Concerning care professionals, inclusion criteria were working in a geriatric facility having implemented a DiTV system, and having been involved, at some level, in its implementation. Six professionals were recruited to take part in the interviews (three socio-cultural animators, one deputy director, and two technical managers).

### 2.3. Materials

#### 2.3.1. The DiTV System

Facilities in this study used the DiTV system e-lioTV [42]. This device, developed by the start-up Technosens^®^ (Grenoble, France) [43], is recently available in a small number of NHs and is accessible directly from each resident’s room. Usually, the installation process is as follows: the facility director, or group decides to purchase the device, then installers come to set it up in the residents’ rooms. In addition to the traditional TV service, the DiTV offers access to some additional services using a single remote control:

- *Video calls*. The user can make video calls directly using the TV. A wide-angle camera is positioned directly on the TV screen which can only be activated during a call via a dedicated button on the remote control. On the family side, both a web and a mobile application, compatible with the DiTV system, are available to receive and make video calls.
- *Messaging and photos*. Using the family application, OAs’ relatives can send to the user electronic messages, including pictures or not. A notification on the DiTV system will alert the user about the reception of the message.
- *News from the institution*. The user can consult the news shared by the facility such as the menu of the day, the schedule of socio-cultural activities, or photos of past activities.
- *Games*. The user has access to several digital games such as quizzes, sudokus, and dictations.
- *Radio*. The user can listen to several radio stations.
- *Home automation*. The user can control several home automation elements, such as shutters or lights, thanks to a dedicated interface on his TV.

**Figure 1:**
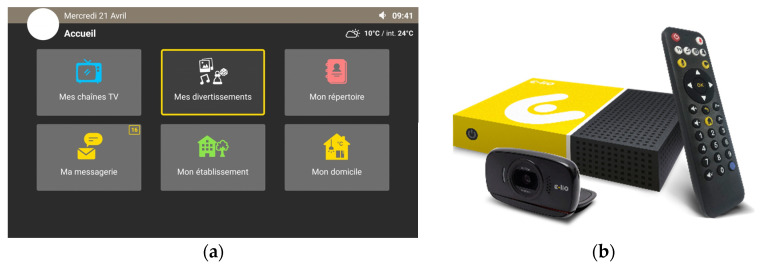
The e-lioTV system: (**a**) TV interface (main menu); (**b**) Box, camera, and remote control.

**Figure 2:**
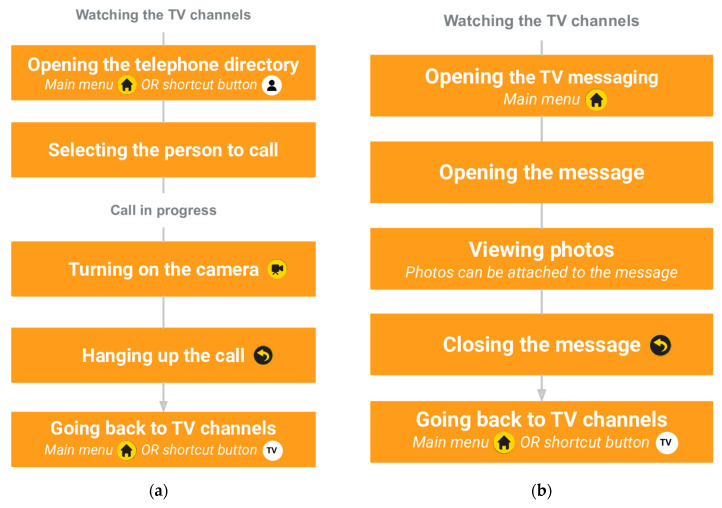
Task diagrams representing the processes for: (**a**) making a video call; (**b**) checking a message on the DiTV.

Those services are available via the main menu (home button on the remote control) or the shortcut buttons (white buttons on top of the remote control). The e-lioTV system is presented in Figure 1. Examples of the mechanisms to access the messaging and the video calling functions are presented in Figure 2.

#### 2.3.2. The Interview Guides

Two interview guides were conceived for this study. The first one aimed at interviewing residents, addressing the following points: (a) Collection of general socio-demographic data; (b) Assessment of the residents’ experience with the training and use of the DiTV; and (c) Identification of barriers to the use of the DiTV system (if applicable). The interview guide for OAs is presented in Table 1.

The second interview guide aimed at interviewing professionals, addressing the following points: (a) Identification of the stages of the DiTV integration in the geriatric institutions (e.g., installation, training, first use), and the role of the professionals during each stage; and (b) Evaluation of the professionals’ satisfaction with the DiTV. The interview guide for professionals is presented in Table 2.

Both interview guides were pre-tested with a few professionals and elderly users. These pre-tests helped to further refine the guides, and to improve the conduct of the interviews to obtain useful data.

### 2.4. Procedure

The case study was structured into two parts. The first one involved interviewing residents from NHs, who had a DiTV installed in their rooms, to explore their perspectives on using the system and the difficulties encountered. On the day of the interviews, the researchers explained to the OAs the objectives of the study, and give them an information letter summarizing the main points of the study and their rights. Finally, all the OAs signed a consent form if they were willing to participate in the study and then, the interview was started. During the interview, the participant was asked to turn on the DiTV system and to show the functionalities used, allowing the researchers to observe, first, the sequence of steps used to access the different functionalities and then, the difficulties encountered with the remote control and the interface (e.g., hesitation in selecting an item on the interface, or the difficulties to press a button on the remote control). Throughout the interview, the participant’s comfort was assured by offering breaks if necessary All the interviews were recorded for data analysis. The second part of the study consisted of semi-structured interviews with professionals with the main objective of understanding the organizational dispositions taken, or required, for the implementation of the DiTV in the facility. The day of the interviews, the researchers scheduled appointments with the NHs’ professionals according to their planning. For these interviews, notes were taken instead of being recorded. The semi-structured interviews were performed by two trained researchers in interview techniques. Ethical approval for this study was obtained from the Research and Ethics Committee held by Université Paris Cité in November 2021 (No. 00012021-91), and validation for the data management and its compliance with the General Data Protection Regulation (GDRP) was registered with the Data Protection Office, in the general register of Greater Paris University Hospitals (AP-HP) processing in February 2022 (No. 20220228123925).

### 2.5. Data Analysis

Only interviews with residents were recorded and transcribed. We carried out 18 interviews which lasted between 8 and 57 min.

The analysis of the transcripts was carried out using the European Health Technology Assessment model (EUnetHTA Core Model^®^, Version 3.0, Diemen, The Netherlands) created by the international expert group of the European Network of Health Technology Assessment (HTA) [36]. Although the main aim of this model is to enable international collaboration in producing HTA information, it can be used for research purposes in the field of health technologies [44]. This model includes nine domains for the assessment of health technologies: 1. Health and Current Use of the Technology (CUR), 2. Description and Technical Characteristics of Technology (TEC), 3. Safety (SAF), 4. Clinical Effectiveness (EFF), 5. Costs and Economic Evaluation (ECO), 6. Ethical Analysis (ETH), 7. Organizational Aspects (ORG), 8. Patient and Social aspects (SOC), and 9. Legal Aspects (LEG). Each domain comprises several topics, and each topic is divided into issues (i.e., generic questions that should be considered when assessing health technology). A domain, topic, and issue combination define a unique assessment element ID. Proper registration of the use of EUnetHTA Core Model^®^, Version 3.0 for this work was made on the HTA Core Model^®^ website [45].

Transcripts were first coded according to the nine domains of the EUnetHTA Core Model^®^ and then reread several times to improve familiarity with the data. During this stage, 18 topics and 22 issues from the EUnetHTA model that were deemed relevant for the study were pre-selected. The coded segments were then classified according to these issues, grouping segments with similar ideas. The coding was conducted by BN and ASR. The MAXQDA software (20.4.0) [46] was used for this purpose.

**Figure 3:**
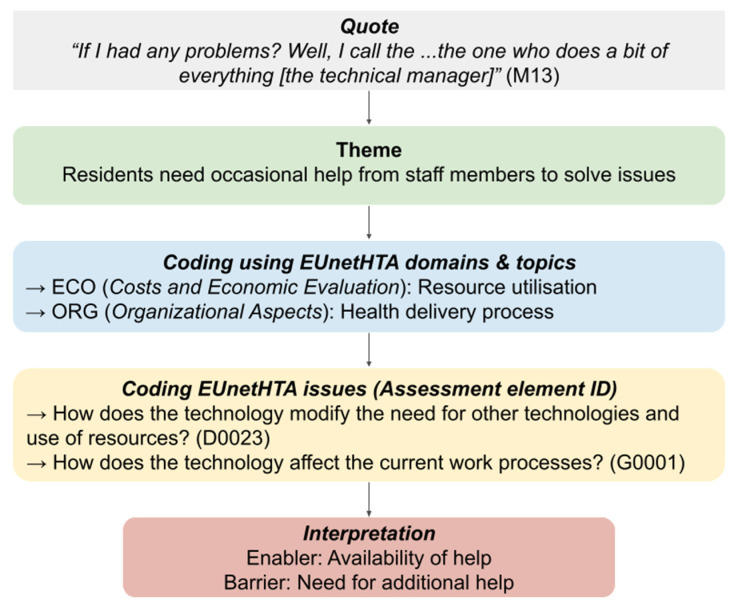
Example of data coding using the EUnetHTA framework.

The interviews with professionals were also analyzed to complete and identify new organizational and technological issues. Key verbatims from professionals were literally transcribed and used to identify or complete existing themes from resident interviews. This process of data source triangulation was used to ensure data validity [47]. Finally, the emerging themes were categorized as enablers, barriers, or findings on the use of the device. Interviews were processed until no new enablers and barriers were identified, reaching data saturation. In total, 17 topics and 21 issues from the EUnetHTA Core Model^®^ were used (Table 3). An illustration of the analysis process is presented in Figure 3.

## 3. Results

**Figure 4:**
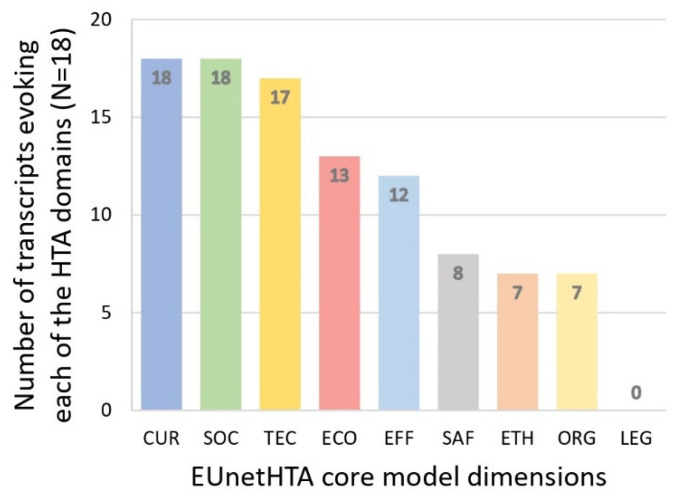
EUnetHTA Core Model^®^ domains occurrences within the 18 transcripts of the OAs’ interviews.

In the following sections, each theme identified in the interviews is associated with one or more EUnetHTA Core Model^®^ domains and element IDs. A summary of the distribution of the nine major EUnetHTA domains (CUR, TEC, SAF, EFF, ECO, ETH, SOC, and LEG) by transcripts is shown in Figure 4. Representative transcript verbatims are presented in some sections. Verbatims were anonymized and are presented as follows. For residents: the “Res” prefix and participant number (1–18); for professionals, the respondent title (deputy director, socio-cultural animator, technical manager), and participant number (1–6, if multiple participants with the same professional title were interviewed) e.g., a verbatim of the socio-cultural animator n°3 would be coded (Socio-cultural animator 3).

### 3.1. Socio-Demographic Data (CUR: A0005)

Of the 18 OAs interviewed, some participants had mild or severe visual impairments (*n* = 4) (e.g., nearsightedness or inability to differentiate the buttons on the remote control), or mild or severe hearing impairments (*n* = 4). Some participants also had motor disorders (*n* = 7) (e.g., difficulty walking, grasping objects, such as fine motor skills), or complained about cognitive disorders (*n* = 6) (e.g., memory, attention). Finally, among the residents interviewed, some participated regularly in the activities (e.g., active and integrated into the facility) (*n* = 12), while others refused to participate in these activities because of voluntary isolation (e.g., did not feel in tune with the NH population, dissatisfaction with the management of the facility, tiredness, or sensory or cognitive disorders restricting the activities). Some participants did not want to communicate their last occupation (*n* = 8), so no analysis could be made on this topic.

### 3.2. Experience with the Use of the DiTV Functionalities (CUR: A0001)

Of the 18 residents interviewed, only 4 did not use the additional DiTV services, i.e., they watched TV channels only. In general, the other 14 residents mostly used the *News from the institution* (*n* = 10), the *Radio* (*n* = 8), followed by *Video calls* (*n* = 7), *Messaging and photos* (*n* = 6), *Home automation* (*n* = 5), and finally *Games* (*n* = 1). It is important to note that the *Home automation* functionality was not available in two NHs.

### 3.3. DiTV Use as a Useful and Feasible Intervention

#### 3.3.1. DiTV Use as a Useful Intervention (EFF: D0012, and D0017; CUR: F0001, and A0001; SOC: H0006, and H0100)

All the participants declared having no expectations regarding the DiTV when they arrived at the facility. However, when the services available on the DiTV were briefly re-explained by the interviewer in this study to OAs who used TV channels only (*n* = 4), two of them mentioned that they expected the video calling functionality of the DiTV would help them to reconnect with relatives abroad or that the game functionality would stimulate their memory: “*Oh, I’d be interested in that [video calls on the DiTV system]. Because my son is in America*.” (Res16); “*[Do you like quizzes?] Yes, yes, it exercises your memory because that’s what you lose first, and sometimes it’s stressful*.” (Res3).

Among the DiTV users (*n* = 14), it was reassuring to find familiar services, such as TV channels and radio stations on their DiTV when they arrived at the facility. In addition, most participants limited their use to these functionalities: “*Apart from TV [channels]… I’m not interested in anything else*.” (Res12).

Beyond these entertainment services, the most used functionalities on the DiTV were the social networking ones (e.g., information about the institution, messages, and video calls). Some residents (*n* = 9) mentioned that social networking services helped them remain connected with their family but also with their institutional environment, allowing them to be better integrated in the facility: “*I use it to communicate with my family, that’s all! My family, and what’s going on in the house, otherwise ... [nothing else]*.” (Res13).

In this respect, in the facilities examined, there was no system in use that would allow residents (or their families) to easily consult the news of the institution. Menus or entertainment programs were generally posted in the corridor or in the lift, which was difficult for some residents in wheelchairs to access: “*But how do you want me to see it [the restaurant menu]? I can’t stand up and sit down I can’t see it*.” (Res18). The DiTV made more accessible this kind of information, strengthening the social link between the OAs, their families, and the institution.

No NHs’ residents evocated the COVID-19 during the interviews. However, the usefulness of the device for OAs and their families during this period, where no visits were allowed in the institutions, was highlighted by a professional: “*Families found e-lioTV very good at first, then with COVID, it became great, even essential*” (Deputy Director 5).

#### 3.3.2. DiTV Use as a Feasible Intervention (TEC: B0001; CUR: F0001, and A0018; ORG: G0010)

The usability and accessibility of the DiTV were critical to facilitate the implementation of the intervention with OAs. The TV interface and the remote control are familiar and reassuring devices for elderly residents. Most of them have already integrated the use of the remote control (*n* = 12) (e.g., increasing/decreasing the volume, changing channels): “*It’s typical!*” (Res16); “*I already had one before*.” (Res18), thus making it easier for them to use the DiTV, provided they have the necessary will and cognitive resources: “*When you’re mentally competent*.” (Res13). Moreover, OAs including those suffering from arthritis considered that the buttons were easy to press: “*No, I’m fine [with the use of the remote control], even though my thumbs hurt*.” (Res2). Regarding the interface, the labels and icons seemed clear and understandable. Finally, compared to other technologies, such as smartphones or touch tablets, the TV interface offered the opportunity to view content on a large screen, thus becoming a more accessible and user-friendly tool.

Another critical aspect for the implementation of the intervention was the professionals’ satisfaction with the DiTV system. Staff members were overall satisfied with the DiTV functionalities as they considered it responds to OAs’ needs of entertainment and social contact (e.g., recreational games within the framework of socio-cultural activities, more opportunities for social contact): “*Video calls are great!*” (Technical Manager 4).

Being able to make video calls directly to the residents’ TV was considered to be a “time saver” for the staff. Compared to the use of a tablet, the staff no longer needed to store an additional device or to go back and forth in the rooms to install it (especially in NHs). Furthermore, with the DiTV system it was possible to leave the resident take his call with his family alone, once the call was in progress. This possibility limited for the professional the need to be present for the entire call, time that could be used to take care of other residents. Therefore, the setup of the DiTV system was simpler and quicker than tablets or other devices for the staff, increasing the adoption of the DiTV by staff members.

Data analysis from the 18 transcripts of residents’ interviews and the 6 professionals’ interviews revealed 4 enablers and 11 barriers to the use of DITV in geriatric settings.

### 3.4. Enablers for the DiTV Intervention in NHs

#### 3.4.1. Training of Staff Members on the Use of the DiTV System (ORG: G0003)

When the DiTV was installed in the institution, experts came to train a few key volunteer staff members (e.g., socio-cultural animator, technical manager, and some care staff members). Sometimes a DiTV system referent person was appointed, being responsible for passing on the knowledge to other staff members when necessary (e.g., turnover, sick leave, etc.). This “system referent” model created a dynamic within the institution around the DiTV system, facilitating the dissemination of information among professionals and residents.

#### 3.4.2. Training of OAs on the Use of the DiTV System (TEC: B0014)

It is only by cross-checking the information from the interviews with professionals and those with residents that we could understand how the training was provided. Especially as the duration and details of the training given were not precisely remembered by the residents. Seven OAs stated they learn to use the DiTV system progressively by themselves, or with the support of their family. However, in most cases, a single training session, rather in a demonstration format, was given to the residents by an animator (*n* = 9), or a caregiver (*n* = 2) when they arrived at the facility. All of them received printed documentation concerning the remote control (e.g., a diagram of the remote control with the role of each button) and the family application. This last support was mainly addressed to the resident’s relatives. Even though the demonstration was not designed as formal training for OAs (e.g., an errorless training with spaced retrieval), some participants reported that they continued to learn on their own or with the help of their families (*n* = 8): “*The first time I was told...that’s it! You can have a video call with your friend...they showed me how, and that was it. [And then you managed on your own?] Oh yes, on my own*.” (Res13).

In one NH, some residents explained that the socio-cultural animator conducted a group workshop to complement the first training, helping to reinforce the knowledge of the residents about the DiTV services.

#### 3.4.3. Availability of Assistance for OAs for the Use of the DiTV System (ECO: D0023; ORG: G0001)

The residents interviewed mentioned the availability of assistance for the use of the DiTV, and recognized its usefulness, particularly in the event of technical problems (intervention of the technical manager, care staff members), or in the event of occasional or recurrent difficulties of use encountered by themselves (intervention of the animator, technical manager, care staff members, or even the family) or their family (intervention of the animator or the technical manager). The residents knew whom to contact in case of problems: “*If I have a problem, I call in a carer*.” (Res14); “*If I had any problems? Well, I call the ...the one who does a bit of everything [the technical manager]*.” (Res13).

#### 3.4.4. OAs’ Interest in Innovative Technologies (CUR: A0018; EFF: D0012; SOC: H0006)

Among the participants, 12 residents had a smartphone (*n* = 7) and/or a tablet (*n* = 9), and/or a computer (*n* = 5). Some of them were interested in technological innovations and showed pride in knowing how to operate the DiTV system. Two residents reported having shown friends and visitors the *Home automation* and *News from the institution* services of the DiTV: “*I sometimes give demonstrations of the home automation to visitors*.” (Res6). Previous interest in the use of digital technologies can be considered as a factor contributing to the motivation to try new technological tools in OAs.

### 3.5. Barriers to the DiTV Intervention in NHs

#### 3.5.1. Loss of Knowledge with Staff Members’ Turnovers (ORG: G0008)

There was not a systematically appointed system referent person for the DiTV in all the institutions. Thus, in the event of recurrent turnover, the skills acquired in using the system were lost and new training had to be provided. However, newcomers did not necessarily request training (e.g., they were not necessarily aware of the DiTV system’s existence), and experts were not necessarily aware of new arrivals. This loss of knowledge, resulting from professional turnover, seemed to hinder the adoption of the DiTV by professionals and therefore by residents.

#### 3.5.2. Ergonomic and Technical Issues in the DiTV System (TEC: B0001; EFF: D0012, and D0017; SAF: C0005)

A second barrier to the use of DiTV was its operation. Some participants experienced technical issues (e.g., TV channels not accessible or poor video quality during calls) (*n* = 8) and ergonomic issues (*n* = 7) when using the DiTV. Not all the participants were observed while using the DiTV during the interviews (e.g., video calls and games). However, the observations conducted helped to identify that the shortcut buttons were generally preferred to the main menu to access some DiTV services (e.g., radio and news from the institution functionalities). A summary of the ergonomic issues is presented in Table 4.

Some participants expressed frustration, annoyance, and anger because of the DiTV’s issues (*n* = 5), exposing them to psychological risks: “*I think everyone was annoyed about the fact that this DiTV doesn’t work*.” (Res17). Indeed, TV channels were often the only means of distraction available to OAs in geriatric facilities. Technical and ergonomic problems tended to negatively affect the user’s perception of the DiTV (e.g., technology does not work well, too complex), influencing its long-term adoption. Another consequence of these problems was a decrease in the OAs’ sense of self-efficacy and their self-esteem, as they felt responsible for the DiTV’s malfunctioning: “*If it doesn’t work [the DiTV system], it’s because we’re making mistakes*.” (Res10).

In addition, the interventions of the facility staff (e.g., technical manager or socio-cultural animator) to solve technical issues represented an additional workload for them, which could contribute to reducing the professionals’ satisfaction with the tool (e.g., regular visits by the technical manager to restart not working televisions).

#### 3.5.3. Inadequate Training Format on the DiTV for OAs (TEC: B0014; SAF: C0005)

The current format of training provided to most OAs consists of a single demonstration: “*It was succinct... [My daughter] had shown us a little bit [how to use the DiTV system] but otherwise..*.” (Res10). However, OAs need repetition and time to assimilate new knowledge. A major drawback of this single demonstration is that OAs often reached cognitive saturation, being confronted with too much information. Thus, among the DiTV users (*n* = 14), 10 participants had only partial knowledge of the services available on the DiTV, preferring to focus on only 1 or 2 functionalities. In other cases, residents did not try the additional services of the DiTV, preferring to give up because they felt that they were too old and not able to use it: “*I’m turning 80 this year, so I’m not used to this kind of exercise [having video calls on TV]*.” (Res17).

#### 3.5.4. Choice of an Inappropriate Timing Both for Introducing the System to OAs and for Training (CUR: A0005; SOC: H0012)

The training for the DiTV use was often carried out when the resident arrived at the institution, or in the weeks that followed. It is worth noting that this moment represents a significant change in OAs’ lives and is a source of stress and anxiety. Moreover, institutionalization in a geriatric establishment often follows a deterioration in OAs’ health or the loss of a loved one. The OAs were therefore tired and not in the best moment to learn to use new technology: “*I told myself we’ll see! Yes, especially when you arrive like that ... After moving here, I was still not in good shape*.” (Res7).

#### 3.5.5. OAs Need Additional Help Using the DiTV System (TEC: B0004; ECO: D0023; ORG: G0001; SOC: H0012)

Among the participants, two residents needed staff members to select the TV channels, while the others were partially or completely independent. Needing to regularly ask for help, in case of technical issues or because of the poor accessibility of the DiTV, could discourage some residents from using the DiTV, who were then hesitant about bothering staff members or relatives: “*Maybe it’s because we don’t ask enough [how to use the DiTV]. Maybe that’s it [the problem]!”* (Res4); *“Yes [I’m interested in video calling services] but someone has to set me up, I can’t do it by myself*.” (Res16).

#### 3.5.6. OAs’ Health Issues (CUR: A0005)

Beyond the accessibility of the technology, sensory, cognitive, and/or motor impairments could limit the use of the remote control, as well as the use of some services on the DiTV. As mentioned above, participants had visual (*n* = 4), hearing (*n* = 4), motor (*n* = 7), and/or complained about cognitive (*n* = 6) impairments.

Finally, these impairments could limit some activities (e.g., reading, writing, sewing), leading to a general discouragement in the OAs: “*I don’t want anything anymore. That’s the big problem*.” (Res16).

#### 3.5.7. OAs’ Low Self-Efficacy (SOC: H0012)

OAs were generally reluctant to explore and navigate the DiTV interface on their own. A single demonstration, in this case, did not seem to be enough to encourage residents to discover the features on their own. Some OAs expressed discomfort and fear of making mistakes or breaking the device (*n* = 4): “*[A resident talking about his wife with whom he shares an accommodation] She is afraid of making a mistake [...] of damaging the device”* (Res6); *“But you know, at this age [beyond 80 years old] ... well, you hesitate [you are less sure of knowing how to do], right?*” (Res4).

#### 3.5.8. Technological and Social Habits of OAs (CUR: A0018, and F0001; SOC: H0012)

Learning to use new technologies can be disruptive and therefore very demanding for frail OAs, especially when they move into the geriatric facility. Furthermore, as mentioned earlier, 12 residents were already equipped with smartphones, tablets, and/or computers. Smartphones were generally used to access applications, such as *WhatsApp* [48], *Facebook* [49], *Messenger* [50], or *FaceTime* [51], while tablets and computers were used to play games, go on YouTube, search the Internet, or view photos online. Four residents had already made the effort to learn how to use new technologies and software, and thus acquired some habits regarding software and applications: “*It’s more convenient on the cell phone [than on the DiTV]! On Facebook, on FaceTime…*” (Res1).

In addition, the residents usually had a social routine with their relatives, as well as established times and days for visits, and telephone calls. These social habits developed by the OAs and their relatives tended to decrease their interest in new services, such as messaging or video calls using the DiTV system, always preferring physical visits: “*I prefer something real*.” (Res17).

#### 3.5.9. Digital Divide (SOC: H0012)

Some OAs reported feeling overwhelmed by technological innovations, considering them too complex or simply not being of their generation (*n* = 4): “*I didn’t follow the evolution [of the technology], it was too fast*.” (Res8).

As they felt not “technological” enough to be interested in the device, two participants abandoned the idea of using the DiTV by themselves and left the “technical” part to their spouses with whom they share their rooms (e.g., setting up the video calls, and searching for the activity schedule): “*I take care of everything concerning the house. And then everything that concerns the technology [the usage of the DiTV system] is always my husband*.” (Res9).

#### 3.5.10. Families’ Interest in DiTV Use Perceived by OAs (SOC: H0012)

Another barrier identified to the use of the DiTV was related to the OAs’ family and friends. Like the resident, the family already had technological and social habits (e.g., use of *WhatsApp* [48] on a mobile phone, or visits). Several residents thought that their family could be too busy to call or send messages, not interested in social networking services, or be more interested in visits than in video calls: “*But I think that they... it [the DiTV system] may not be what they like best either. A phone call is more convenient, I think*.” (Res4). In all cases, the family seems to be a key driving force for the resident’s use of the DiTV.

#### 3.5.11. Ethical Questions Raised by the Implementation and Use of the DiTV System in Geriatric Settings (ETH: F0101, F0011, and F0004)

Finally, the multidimensional analysis allowed the identification of four ethical barriers to the use of DiTV in geriatric settings.

First, regarding the video calling and messaging services, three participants mentioned privacy as a reason for not using the DiTV. For example, one resident did not want to make video calls because she did not want to be seen in her current physical condition (although the camera is only activated at the resident’s request): “*First of all, I don’t like it, having a video call when my hair is not done*.” (Res17). The use of these services could therefore sometimes be perceived by OAs as an intrusion into their private life.

Secondly, the resident’s relatives can see the activities carried out in the facility, the photos, and therefore who took part in the activities or not via the family application. The resident may then feel monitored, raising the question of privacy and user control over it: “*Sometimes they [the resident’s children] call me, and they are more aware than me! [about the activities in the institution]*.” (Res2); “*My son is registered on the site [the family application], so he watches, and he tells me: Where were you? What were you doing?*” (Res3).

Thirdly, one resident noted a decrease over time in the number of messages and video calls from their relatives received through the DITV system for which they had no explanation. The advantages of social networking services can then turn into disadvantages, which can cause the resident to feel abandoned: “*Well, it happened yes [to receive messages]... But I have less [messages] now, at the beginning I had a little more..*.” (Res4).

Finally, although technology offers many possibilities, one resident reported feeling forced to learn how to use this new TV: “*[Interested in the tool?] Yes... although... because we’re forced to! We better get started [to learn how to use the DiTV]*.” (Res8). This could be explained by the fact that in the facilities examined, the DiTV was not installed according to the will of the residents, on a case-by-case basis, but as the result of a collective decision made by the institution or the group. This procedure may raise questions about the participation of the resident in the decision of whether to use the tools that are proposed to him/her in the institution.

A summary of the 4 enablers and 11 barriers identified regarding the use of the DITV system in geriatric facilities is presented in Table 5.

## 4. Discussion

The purpose of this research was to study the enablers and barriers of an intervention involving the implementation and use of a DiTV system in NHs, using the example of e-lioTV. Three geriatric institutions participated in our research, and 18 users (OAs) and 6 professionals were interviewed concerning factors that determine the use of a DiTV. The analysis was performed using the EUnetHTA multidimensional framework [36]. This methodology allowed us to expand our analysis beyond the users’ characteristics and the devices’ ergonomic by considering the geriatric institution environment and more general aspects, such as ethical or economical aspects. The analysis showed that the use of the DiTV is limited by organizational (e.g., workload), technological (e.g., ergonomic issues), human (e.g., health issues), ethical (e.g., privacy), and safety factors (e.g., frustration due to technical problems).

### 4.1. DiTV Use as an Intervention in NHs

Among the participants in the study, 14 found the DiTV useful for entertainment or staying connected with their family and institution, and 12 found it accessible and easy to use. Such technology could help OAs strengthen their social link with their environment, thus reducing social isolation and loneliness in NHs.

Although results of interventions involving ICTs are promising, there are still few studies that evaluate long-term benefits. Thus, no significant evidence was found to support the effectiveness of ICTs on reducing loneliness and social isolation in OAs [37,52]. However, the efficacy of the interventions could be better assessed by involving larger sample sizes, and providing training and support to the facilitators or coordinators of the interventions [37]. This last observation is in line with the results of this study.

### 4.2. Factors of DiTV Use and Acceptance

The results obtained in this case study, with OAs living in geriatric institutions, seemed in line with the grounded model about technology use elaborated by Chen et Chan [53]. In their qualitative study about gerontechnology use by OAs living at home, the authors showed that the use of gerontechnology is determined by three outer layers: (1) the personal (e.g., attitudes toward technology, individuals’ attitudes, and self-perceptions about oneself, functional capacity, and knowledge), (2) the technological (e.g., utilitarian, social, hedonistic outcomes, usability, and safety), and (3) the environmental context (e.g., social influences, training, assistance and encouragement, and situational characteristics such as time and accessibility). In this section, we focus on the issues resulting from the interaction between the personal and the technological context, the technological and environmental context, and the interaction between the two stakeholders who compose the environmental context. Finally, we look at some limitations of the study, providing suggestions for future work.

#### 4.2.1. Interactions between the Personal and the Technological Context

Among the issues resulting from the interaction between the personal and the technological context [53], some OAs expressed an interest in innovative technologies, most of them (*n* = 11/18) owning already a smartphone, a computer, or a tablet. This interest could reflect a good computer self-efficacy, encouraging the adoption of the DiTV system. However, the digital divide remains a barrier to the use of a DiTV system, even though TV support is well-known in this population. OAs who did not own a computer, tablet, or smartphone (*n* = 7/18) were less accustomed to technological innovations, feeling more distrustful about new devices in general.

In this case study, the DiTV system studied failed to enhance the usability of a video calling interface, creating on the contrary new ergonomic issues. Carvalho et al. [54] conducted a literature review about the barriers to the use of TVs, and more specifically of remote controls, by OAs. According to them, the problems encountered come mainly from individuals’ characteristics (e.g., trembling hands, grasping issues, finger imprecision, visual impairments, memory decrease, learnability decrease), but also from the ergonomics of devices, particularly the remote control (e.g., including a large number of functions, unfamiliar functions, small labels, small symbols, small buttons, a small gap between buttons, inflexible layout (e.g., buttons cannot be reorganized), small device, lack of a backlight, requiring a specific body position to avoid losing infrared signal). This electronic device, which is already part of the everyday life of OAs [55], needs to be adapted to the rapid development of new services accessible through the TV interface.

In the case of the DiTV system, the addition of a few services to the traditional TV channels makes the interface and the use of the remote control more complex (e.g., by adding lists, menus, and non-linear navigation on the TV screen), raising some questions regarding their use by OAs: Is the interface simple enough? Are all the features useful? Is the remote control adapted to the interface and OAs? Are there enough buttons or too many buttons? It was also observed that the vocabulary used in the DiTV interface might be too technical for OAs and the web-based navigation not adapted to this type of display and remote control.

The mixed results obtained in the study are in line with the literature [26]. TV support offers a real opportunity in terms of accessibility and usability for OAs, but work remains to be done to adapt the remote control and the interface to this new use of TV. Castilla et al. [56] recommend using linear navigation when designing interfaces for OAs. They seem to prefer this type of navigation, enhancing their success rate and lowering their performance time.

Other solutions have been put forward ranging from the use of a simplified remote control, or a tablet, to the use of voice commands or gestures, changing the interaction paradigm at the same time [57]. Voice interaction has been already studied a few times in the literature as shown by Carvalho et al. [54], and seems to be an interesting solution to make the DiTV system more accessible. A few authors have synthesized design guidelines for OAs and TVs [31,54] or OAs and displays [56,57,58]. However, those studies have not yet been conducted in geriatric settings, where the population and environment may create other issues (e.g., intimacy issues, talking to technology could not be socially acceptable, vocal interaction is not reliable for OAs, etc.) [26,59]. More detailed and applied recommendations need to be formulated through extensive user-centered techniques. Task analysis could be an interesting method to identify the specific cognitive, perceptual, and motor demands, as well as the necessary information required to complete successfully a video call, or a messaging task with the DiTV [57,60].

#### 4.2.2. Interactions between the Technological and the Environmental Context

Concerning the factors influencing the DiTV system use and adoption related to the interaction between the technological and the environmental context [53], the literature shows that family members can be both a source of motivation but also a barrier to technology use in OAs [61]. In the case of the DiTV system, services such as messaging, or video calls depend on the use of another web and smartphone application for family members. According to the interviews, some residents have seen their number of messages and/or calls decrease over time. This decrease could be linked to the presence of technical and/or usability issues in the family application. This hypothesis remains to be verified in a study dedicated to this application and its use by families. Moreover, OAs’ family members include several generations, with diverse experiences and knowledge in terms of technology. User profiles could allow us to identify the expectations, needs, and feelings of OAs’ families regarding the technology, or the institutionalization of their relative, and to determine the functional specifications of different categories of family users [62].

#### 4.2.3. Interactions inside the Environmental Context

Finally, the interviews did not allow for exploring how the interaction between the family and the institution, the two stakeholders of the environmental context of OAs [53], influenced the use of DiTV. The role of the resident’s relatives is indeed complex, since they usually remain involved in their caregiving role, even after the institutionalization [63]. Admission to a geriatric institution is a turning point for relatives, accompanied most of the time by a transfer of certain responsibilities from the family to the care staff. Families often experience loss, guilt, and shame but also feelings of relief [64]. Family involvement in care interventions, once the OA is admitted to an institution, can be problematic for many reasons (e.g., poor interpersonal relations with staff members, reluctance of families to be involved, resistance to change, etc.). In some cases, staff members perceive family members as barely involved in the care process, while family members perceive staff members as reluctant to their involvement [63]. Haesler and al. identified six factors that are essential to the development of constructive family-caregiver relationships: upholding the patient’s uniqueness; assessing and addressing individual family needs; using effective communication skills; implementing a collaborative care process; understanding and addressing interpersonal power issues; and providing organizational support [63].

The implementation of the DiTV requires the use of a TV by the resident, the sharing of contents by the facility, and the sending of messages and making of video calls by the family. However, the family can only fulfill its role if the institution provides them with their login and all the information regarding the DiTV functioning. Thus, the family’s supportive role depends partly on the quality of the collaboration with the institution. A more detailed study about the link between the DiTV system use and the family-institution relationship could identify other conditions that guarantee the effective use of this device.

### 4.3. Limitations of the Study

This case study had several limitations, such as the small number of interviews conducted. The small sample in the study prevented us from studying factors that could help to better understand the use and adoption of new technologies, such as the DiTV, by OAs. This is the case of specific socio-demographic characteristics, such as the educational and professional background. Furthermore, because of the lack of a control group, we cannot conclude if our sample was representative of the NH population, particularly regarding technology acceptance and digital literacy. Because of the small number of participating institutions, it was not possible to take into consideration NHs organizational information for the analysis.

Another limitation was the absence of the resident’s entourage in the interviews, though they are part of the environmental context of the DiTV system use. The family-facility relationship and its impact on the use of the device were not explored.

Finally, the information about DiTV use was sometimes incomplete or biased, obtained only from the facilities and the residents’ perspectives (e.g., the frequency of video calls, the residents’ perceived motivation of families, etc.). It was indeed difficult for OAs, with potential mild to moderate cognitive impairment, to verbalize all the difficulties they encounter when using the DiTV system. Thus, the understanding of DiTV learning and use within geriatric settings was sometimes incomplete.

## 5. Recommendations for the Implementation of a DiTV in Geriatric Settings

### 5.1. To Identify a Professional Reference Group for the DiTV System within the Facility

The system referent is the privileged contact of the DiTV supplier. Its role is to centralize and transmit to the experts the requests or issues related to the DiTV but also to train the newly arrived professionals. To reduce this workload, it would be interesting to identify a small group of referents rather than a single one. This would have the advantage of dividing responsibilities and sharing the workload associated with the gestion of the DiTV intervention. Ideally, one of the members of the group should be the technical manager, as this person usually has more technical knowledge than the other professionals in a geriatric facility.

### 5.2. Allow Residents to Be More Autonomous in the Choice and Use of Entertainment and Communication Systems

In geriatric institutions, the daily lives of residents are shaped by structured routines, primarily designed to provide efficient care with the resources available. These routines may limit the freedom of some residents by reducing the control they can have over their environment and their activities [65]. To preserve the physical and mental health of residents, Kane et al. [65] recommend, among other things, giving the residents choice and control over telephone and e-mail use. Similarly, it would be advisable to invite residents, who have the capacity to do so, to choose the kind of TV system that they want to use (e.g., DiTV or traditional TV set), and to decide when and how to use it.

### 5.3. Design an Adapted Training for the Use of the DiTV System

A solution to train OAs in the use of new technologies is the use of errorless training with spaced retrieval and fading techniques [66,67]. This method consists of avoiding erroneous responses when learning new information, while decreasing the number of cues provided until the desired response is given without assistance. The information is then repeated and recalled at increasingly long intervals until the desired response is memorized.

The training program should first consist of the presentation of the device (e.g., the screen, the camera, the remote control) to reassure residents about the DiTV. This presentation should explain that DiTV allows for watching TV channels in the same way as usual, and to access other services. Then, depending on the resident’s interest and abilities, one or more services could be presented to them, making sure to respect the good practices of the training for OAs.

### 5.4. Enhance the DiTV Usability and Accessibility

To improve the usability and accessibility of the DiTV, it would be pertinent to adapt not only the design of the interface and the remote control but also the interaction paradigm, i.e., the way the user is expected to interact with the DiTV. In this respect, voice interaction seems to be an alternative, or at least a complement to the standard remote control, to make the DiTV system use more accessible. However, the usability of voice interaction for a DiTV system remains to be studied for the use of more complex services, such as video calls or messages. It would then be interesting to test this new interaction paradigm with a population of OAs living in a geriatric institution.

### 5.5. Involve Families in the Process of Choice, Training, Configuration, and Use of the DiTV Device

During the pre-admission visit, it would be interesting to identify the families who could benefit from DiTV’s social link services (e.g., relatives who live far away or abroad). Support should then be offered to interested families through, for example, occasional workshops with families and residents to train them together on the DiTV system and the family application. Regular family groups could also be used to answer questions from families regarding the use of the DiTV services in the event of problems or other requests. The objective would be to create a dynamic process around the DiTV services.

## 6. Conclusions

The case study aimed to investigate the barriers and facilitators to an intervention involving DiTV use in NHs, using the example of e-lioTV. Based on the EUnetHTA multidimensional model, four facilitators (training of staff members on the use of the DiTV system, training of OAs on the use of the DiTV system, availability of assistance for OAs for the use of the DiTV system, and OAs’ interest in innovative technologies) and 11 barriers (loss of knowledge with staff members’ turnovers, ergonomic and technical issues in the DiTV system, inadequate training format on the DiTV for OAs, choice of an inappropriate timing both for introducing the system to OAs and for training, OAs need additional help using the DiTV system, OAs’ health issues, OAs’ low self-efficacy, technological and social habits of OAs, digital divide, families’ interest in DiTV use perceived by OAs, and ethical questions raised by the implementation and use of the DiTV system in geriatric settings) to the use of the DiTV were identified.

To conclude, the DiTV system was useful for most of NHs’ residents. The use of a television support seems to facilitate the daily life of NHs’ professionals by making it easier for NHs’ residents to access messages and video calls. However, its usefulness was hampered by technical, organizational, and training issues. Thus, five recommendations have been formulated for the implementation of an intervention involving the use of DiTV in geriatric settings.

## Figures and Tables

**Table 1:** Interview guide used for semi-structured interviews with residents.

Theme	Question
Socio-demographic data	Gender
	Age
	What was your last professional occupation?
	Have you got a tablet, a smartphone, or a computer? If so, what do you do with it (them)?
	How do you communicate with your relatives?
	Do you participate in socio-cultural activities organized by the institution?
Experience with the use of the DiTV system	What kind of activities do you perform with your television? How often? Can you show me how do you do to access these functionalities?
	What is (are) the main reason(s) for using the DiTV system?
Training for the use of the DiTV system	How did you learn to use it? What were the difficulties during the learning stage?
Barriers to the use of the DiTV system	Have you experienced any difficulties to use the system? (e.g., problems pressing/finding the right remote-control buttons, technical problems, or perceptual issues) What is the reason(s) for not using the other DiTV services?

**Table 2:** Interview guide used for semi-structured interviews with professionals.

Theme	Question
DiTV implementation	When is the DiTV presented to the residents? (e.g., during the pre-admission visit, the move-in, or before the first video call)
	What role do you have regarding the DiTV system use? (e.g., punctual assistance, trainer, or other)
Training for the use of the DiTV system	Are the residents trained to use the DiTV system? (If applicable) When and how the residents are trained?
Satisfaction	Are you satisfied with the DiTV functionalities?
	Are the residents satisfied with the DiTV functionalities?

**Table 3:** Summary of the 21 EUnetHTA Core Model issues selected.

Domain	Topic	Issue	Assessment Element ID
CUR	Target population	What are the symptoms and the burden of disease or health condition for the patient?	A0005
	Current management of the condition	What are the other typical or common alternatives to the current technology?	A0018
	Utilization	For which health conditions and populations, and for what purposes is the technology used?	A0001
		Is the technology a new, innovative mode of care, an add-on to, or modification of a standard mode of care, or a replacement of a standard mode of care?	F0001
TEC	Features of the technology	What is this technology and the comparator(s)?	B0001
		Who administers the technology and the comparator(s) and in what context and level of care are they provided?	B0004
	Training and information required	What kind of training resources and information should be provided to the patient who uses the technology, or for his family?	B0014
SAF	Patient safety	What are the susceptible patient groups that are more likely to be harmed through the use of the technology?	C0005
EFF	Health-related quality of life	What is the effect of the technology on generic health-related quality of life?	D0012
	Patient satisfaction	Were patients satisfied with the technology?	D0017
ECO	Resource utilization	How does the technology modify the need for other technologies and use of resources?	D0023
ETH	Respect for persons	Does the technology invade the sphere of privacy of the patient/user?	F0101
	Autonomy	Does the implementation or use of the technology affect the patient’s capability and possibility to exercise autonomy?	F0004
	Benefit-harm balance	What are the benefits and harms of the technology for relatives, other patients, organizations, commercial entities, society, etc.?	F0011
ORG	Health delivery process	How does the technology affect the current work processes?	G0001
		What kind of process ensures proper education and training of staff?	G0003
	Management	What management problems and opportunities are attached to the technology?	G0008
	Culture	How is the technology accepted?	G0010
SOC	Patients’ perspectives	What expectations and wishes do patients have with regard to the technology and what do they expect to gain from the technology?	H0100
		How do patients perceive the technology under assessment?	H0006
	Social group aspects	Are there factors that could prevent a group or person from gaining access to the technology?	H0012
LEG	No legal aspects		

**Table 4:** Summary of the ergonomic issues reported by residents or observed by researchers during the interviews.

DiTV Functionality	Ergonomic Issues
Main menu	In case of error, OAs have trouble understanding the main menu (i.e., what it is, where they are, and how to go back to the TV channels). Navigation on the main menu is difficult for OAs (e.g., the use of the arrow buttons on the remote control is unfamiliar; experiencing trouble in understanding the item selection).
Radio	Difficulties in understanding the navigation in the menu list (e.g., the possibility to go up and down to choose the radio station)
News from the institution	The text is sometimes too small (for shared contents) and screen glare disturbs the reading. The guidance function sometimes hides some content details (e.g., the schedule of an activity). The icon for the functionality of the shortcut button is not understandable. The shortcut button is too small and is too close to other shortcut buttons.
Video calls	If the resident is absent, the DiTV stays turned on after an incoming call. The volume during a call on the DiTV is too low for OAs (bad signal quality and the TV is a few meters away). The yellow color of the camera button is difficult to perceive for OAs, thus the icon is hardly visible. The camera is on top of the TV screen, thus hardly reachable to readjust its position and angle.
TV channels	The volume (+) and (−) buttons are not physically distinguishable and are too close to each other. Channel buttons are too sensitive (i.e., prolonged pressure on channel 1 results in channel 111).

**Table 5:** Summary of enablers and barriers to the DiTV system use and associated HTA dimensions.

Enablers	Barriers
Training of staff members on the use of the DiTV system (ORG)	Loss of knowledge with staff members’ turnovers (ORG)
Training of OAs on the use of the DiTV system (TEC)	Ergonomic and technical issues in the DiTV system (TEC, EFF, SAF)
Availability of assistance for OAs for the use of the DiTV system (ECO, ORG)	Inadequate training format on the DiTV for OAs (TEC, SAF)
OAs’ interest in innovative technologies (CUR, EFF, SOC)	Choice of an inappropriate timing both for introducing the system to OAs and for training (CUR, SOC)
	OAs need additional help using the DiTV system (TEC, ECO, ORG, SOC)
	OAs’ health issues (CUR)
	OAs’ low self-efficacy (SOC)
	Technological and social habits of OAs (CUR, SOC)
	Digital divide (SOC)
	Families’ interest in DiTV use perceived by OAs (SOC)
	Ethical questions raised by the implementation and use of the DiTV system in geriatric settings (ETH)

## Data Availability

The data presented in this study are not publicly available due to privacy regulations. For more information about the interviews and transcripts, please contact the corresponding author.

## References

[1] Smith D.B.D., Meshkati N., Robertson M.M. (1993). The older driver and passenger. Automotive Ergonomics.

[2] Matthews G., Davies D.R., Stammers R.B., Westerman S.J. (2000). Human Performance: Cognition, Stress, and Individual Differences.

[3] Drageset J., Kirkevold M., Espehaug B. (2011). Loneliness and social support among nursing home residents without cognitive impairment: A questionnaire survey. Int. J. Nurs. Stud..

[4] Nyqvist F., Cattan M., Andersson L., Forsman A.K., Gustafson Y. (2013). Social capital and loneliness among the very old living at home and in institutional settings: A comparative study. J. Aging Health.

[5] Quan N.G., Lohman M.C., Resciniti N.V., Friedman D.B. (2019). A systematic review of interventions for loneliness among older adults living in long-term care facilities. Aging Ment. Health.

[6] Bouillon-Minois J.-B., Lahaye C., Dutheil F. (2020). Coronavirus and quarantine: Will we sacrifice our elderly to protect them?. Arch. Gerontol. Geriatr..

[7] Freedman V.A., Hu M., Kasper J.D. (2021). Changes in older adults’ social contact during the COVID-19 pandemic. J. Gerontol. Ser. B.

[8] Berg-Weger M., Morley J.E. (2020). Loneliness in old age: An unaddressed health problem. J. Nutr. Health Aging.

[9] Latikka R., Rubio-Hernández R., Lohan E.S., Rantala J., Fernández F.N., Laitinen A., Oksanen A. (2021). Older adults’ loneliness, social isolation, and physical information and communication technology in the era of ambient assisted living: A systematic literature review. J. Med. Internet Res..

[10] Khosravi P., Ghapanchi A.H. (2016). Investigating the effectiveness of technologies applied to assist seniors: A systematic literature review. Int. J. Med. Inform..

[11] Döring N., Conde M., Brandenburg K., Broll W., Gross H.-M., Werner S., Raake A. (2022). Can communication technologies reduce loneliness and social isolation in older people? A scoping review of reviews. Int. J. Environ. Res. Public Health.

[12] Verna V., De Bartolo D., Iosa M., Fadda L., Pinto G., Caltagirone C., De Angelis S., Tramontano M. (2020). Te.M.P.O., an app for using temporal musical mismatch in post-stroke neurorehabilitation: A preliminary randomized controlled study. Neurorehabilitation.

[13] De Bartolo D., Morone G., Lupo A., Aloise F., Baricich A., Di Francesco D., Calderone C., Cisari C., Verdecchia G., Sandrini G. (2018). From paper to informatics: The Post Soft Care-App, an easy-to-use and fast tool to help therapists identify unmet needs in stroke patients. Funct. Neurol..

[14] Paiva J.O.V., Andrade R.M.C., de Oliveira P.A.M., Duarte P., Santos I.S., Evangelista A.L.D.P., Theophilo R.L., de Andrade L.O.M., Barreto I.C.D.H.C. (2020). Mobile applications for elderly healthcare: A systematic mapping. PLoS ONE.

[15] Mantovani E., Turnheim B., Domínguez-Rué E., Nierling L. (2018). Navigating the european landscape of ageing and ict: Policy, governance, and the role of ethics. Science Studies.

[16] World Health Organization (2017). Integrated Care for Older People: Guidelines on Community-Level Interventions to Manage Declines in Intrinsic Capacity.

[17] Wang C.-H., Wu C.-L. (2021). Bridging the digital divide: The smart TV as a platform for digital literacy among the elderly. Behav. Inf. Technol..

[18] United Nations Economic Commission for Europe (2021). Ageing in the Digital Era.

[19] Rodríguez M.D., Gonzalez V.M., Favela J., Santana P.C. (2009). Home-based communication system for older adults and their remote family. Comput. Hum. Behav..

[20] Johnson R., Kent S. (2007). Designing universal access: Web-applications for the elderly and disabled. Cogn. Technol. Work.

[21] Cotten S.R., Yost E.A., Berkowsky R.W., Winstead V., Anderson W.A. (2017). Designing Technology Training for Older Adults in Continuing Care Retirement Communities.

[22] Bandura A. (1997). Self-Efficacy: The Exercise of Control.

[23] Davis F.D. (1989). Perceived usefulness, perceived ease of use, and user acceptance of information technology. MIS Q..

[24] Coelho J., Guerreiro T., Duarte C. (2013). Designing tv interaction for the elderly: A case study of the design for all approach. A Multimodal End-2-End Approach to Accessible Computing.

[25] Silva T., Abreu J., Antunes M., Almeida P., Silva V., Santinha G. (2016). +tv4e: Interactive television as a support to push information about social services to the elderly. Procedia Comput. Sci..

[26] Epelde G., Valencia X., Carrasco E., Posada J., Abascal J., Diaz-Orueta U., Zinnikus I., Husodo-Schulz C. (2013). Providing universally accessible interactive services through TV sets: Implementation and validation with elderly users. Multimed Tools Appl..

[27] Mitchell V., Nicolle C., Maguire M., Boyle H. (2007). Web-based interactive TV services for older users. Gerontechnology.

[28] Santana-Mancilla P., Anido-Rifón L. (2017). The technology acceptance of a tv platform for the elderly living alone or in public nursing homes. Int. J. Environ. Res. Public Health.

[29] Zamir S., Hennessy C.H., Taylor A.H., Jones R.B. (2018). Video-calls to reduce loneliness and social isolation within care environments for older people: An implementation study using collaborative action research. BMC Geriatr..

[30] Dou J., Qin J., Wang Q., Zhao Q. (2019). Identification of usability problems and requirements of elderly Chinese users for smart TV interactions. Behav. Inf. Technol..

[31] Ahmed B.S., Bures M., Arai K., Bhatia R., Kapoor S. (2019). Testing of smart tv applications: Key ingredients, challenges and proposed solutions. Proceedings of the Future Technologies Conference (FTC) 2018.

[32] Rivas-Costa C., Anido-Rifon L., Fernandez-Iglesias M.J. (2017). An open architecture to support social and health services in a smart tv environment. IEEE J. Biomed. Health Inform..

[33] Andreadis A., Zambon R., Parlangeli O. (2021). TV as an experience conveyer for better acceptance of ICT services by older adults. Univers. Access Inf. Soc..

[34] Zamir S., Hennessy C., Taylor A., Jones R. (2020). Intergroup ‘skype’ quiz sessions in care homes to reduce loneliness and social isolation in older people. Geriatrics.

[35] Tsai H.-H., Tsai Y.-F. (2011). Changes in Depressive Symptoms, Social Support, and Loneliness Over 1 Year After a Minimum 3-Month Videoconference Program for Older Nursing Home Residents. J. Med. Internet Res..

[36] HTA Core Model^®^ Online|Model. http://www.htacoremodel.info/BrowseModel.aspx.

[37] Findlay R.A. (2003). Interventions to reduce social isolation amongst older people: Where is the evidence?. Ageing Soc..

[38] Khosravi P., Rezvani A., Wiewiora A. (2016). The impact of technology on older adults’ social isolation. Comput. Hum. Behav..

[39] Masi C.M., Chen H.-Y., Hawkley L.C., Cacioppo J.T. (2011). A meta-analysis of interventions to reduce loneliness. Pers. Soc. Psychol. Rev..

[40] Baunstrup M., Larsen L.B., Stephanidis C., Antona M. (2013). Elderly’s barriers and requirements for interactive tv. Universal Access in Human-Computer Interaction.

[41] Opdenakker R. (2006). Advantages and disadvantages of four interview techniques in qualitative research. Forum Qual. Soz. Forum Qual. Soc. Res..

[42] Digital Tools for Seniors for Care Facility Administrators. https://www.e-lio.fr/?lang=en.

[43] Digital Solutions for Nursing Homes and Senior Housing Facilities. https://www.technosens.fr/?lang=en.

[44] Goodman C.S., Ahn R. (1999). Methodological approaches of health technology assessment. Int. J. Med. Inform..

[45] HTA Core Model^®^ Online|Register Use 2021. https://www.htacoremodel.info/RegisterUse.aspx.

[46] MAXQDA|All-In-One Tool for Qualitative Data Analysis & Mixed Methods. https://www.maxqda.com/homepage-2.

[47] Carter N., Bryant-Lukosius D., DiCenso A., Blythe J., Neville A.J. (2014). The use of triangulation in qualitative research. Oncol. Nurs. Forum.

[48] WhatsApp. https://www.whatsapp.com/.

[49] Facebook-Connexion ou Inscription. https://fr-fr.facebook.com/.

[50] Messenger. https://www.messenger.com/.

[51] FaceTime. https://apps.apple.com/fr/app/facetime/id1110145091.

[52] Shah S.G.S., Nogueras D., van Woerden H.C., Kiparoglou V. (2021). Evaluation of the effectiveness of digital technology interventions to reduce loneliness in older adults: Systematic review and meta-analysis. J. Med. Internet Res..

[53] Chen K., Chan A. (2013). Use or non-use of gerontechnology: A qualitative study. Int. J. Environ. Res. Public Health.

[54] Carvalho D., Silva T., Abreu J., Abásolo M.J., Abreu J., Almeida P., Silva T. (2021). Tv remote control and older adults: A systematic literature review. Applications and Usability of Interactive TV.

[55] Oliveira A.P., Vairinhos M., Mealha Ó., Abásolo M.J., Abreu J., Almeida P., Silva T. (2018). Proposal of a tangible interface to enhance seniors’ tv experience: Ux evaluation of SIX. Applications and Usability of Interactive Television.

[56] Castilla D., Garcia-Palacios A., Miralles I., Breton-Lopez J., Parra E., Rodriguez-Berges S., Botella C. (2016). Effect of Web navigation style in elderly users. Comput. Hum. Behav..

[57] McLaughlin A., Pak R. (2020). Designing Displays for Older Adults.

[58] Castilla D., Suso-Ribera C., Zaragoza I., Garcia-Palacios A., Botella C. (2020). Designing icts for users with mild cognitive impairment: A usability study. Int. J. Environ. Res. Public Health.

[59] Cowan B.R., Pantidi N., Coyle D., Morrissey K., Clarke P., Al-Shehri S., Earley D., Bandeira N. (2017). “What can i help you with?”: Infrequent users’ experiences of intelligent personal assistants. Proceedings of the 19th International Conference on Human-Computer Interaction with Mobile Devices and Services.

[60] Hackos J.T., Redish J.C., Redish J.C. (1998). User and Task Analysis for Interface Design.

[61] Luijkx K., Peek S., Wouters E. (2015). “Grandma, you should do it—it’s cool” older adults and the role of family members in their acceptance of technology. Int. J. Environ. Res. Public Health.

[62] Kuniavsky M. (2003). Observing the User Experience: A Practitioner’s Guide to User Research.

[63] Haesler E., Bauer M., Nay R. (2007). Staff–family relationships in the care of older people: A report on a systematic review. Res. Nurs. Health.

[64] Graneheim U.H., Johansson A., Lindgren B.-M. (2014). Family caregivers’ experiences of relinquishing the care of a person with dementia to a nursing home: Insights from a meta-ethnographic study. Scand. J. Caring Sci..

[65] Kane R.A., Caplan A.L., Urv-Wong E.K., Freeman I.C., Aroskar M.A., Finch M. (1997). Everyday matters in the lives of nursing home residents: Wish for and perception of choice and control. J. Am. Geriatr. Soc..

[66] Czaja S.J., Sharit J. (2012). Designing Training and Instructional Programs for Older Adults.

[67] Quillion-Dupré L. (2018). Usage de la Tablette Tactile par les Personnes Agées: Une Approche Ecologique de L’évaluation et de L’aide à L’apprentissage. Ph.D. Thesis.

